# Analytical Investigation of Phthalates and Heavy Metals in Edible Ice from Vending Machines Connected to the Italian Water Supply

**DOI:** 10.3390/foods13182910

**Published:** 2024-09-13

**Authors:** Nicoletta De Vietro, Francesco Triggiano, Pietro Cotugno, Jolanda Palmisani, Alessia Di Gilio, Carlo Zambonin, Gianluigi de Gennaro, Giovanna Mancini, Antonella Maria Aresta, Giusy Diella, Vincenzo Marcotrigiano, Giovanni Trifone Sorrenti, Piersaverio Marzocca, Michele Lampedecchia, Domenico Pio Sorrenti, Ezio D’Aniello, Matilde Gramegna, Alessandra Nencha, Antonio Caputo, Marta Giovine, Caterina Spinelli, Giuseppina Caggiano

**Affiliations:** 1 Department of Biosciences, Biotechnologies and Environment, University of Bari “Aldo Moro”, Via Orabona 4, 70126 Bari, Italy; jolanda.palmisani@uniba.it (J.P.); alessia.digilio@uniba.it (A.D.G.); carlo.zambonin@uniba.it (C.Z.); gianluigi.degennaro@uniba.it (G.d.G.); g.mancini1@studenti.uniba.it (G.M.); antonellamaria.aresta@uniba.it (A.M.A.); 2 Interdisciplinary Department of Medicine-Hygiene Section, University of Bari “Aldo Moro”, Piazza G. Cesare 11, 70124 Bari, Italy; francesco.triggiano@uniba.it (F.T.); giuseppina.caggiano@uniba.it (G.C.); 3 Department of Chemistry, University of Bari “Aldo Moro”, Via Orabona 4, 70126 Bari, Italy; pietro.cotugno@uniba.it; 4 Prevention Department, Local Health Authority “ULSS 1 Dolomiti”, Viale Europa 22, 32100 Belluno, Italy; vincenzo.marcotrigiano@aulss1.veneto.it; 5 Prevention Department, Food Hygiene and Nutrition Service, Local Health Unit BT, Barletta-Andria-Trani, Via Fornaci 201, 76125 Trani, Italy; giovannitrifone.sorrenti@uniba.it (G.T.S.); piersaverio.marzocca@asl.bari.it (P.M.); michele.lampedecchia@asl.bat.it (M.L.); domenicosorrenti@outlook.it (D.P.S.); 6 Prevention Department, Food Hygiene and Nutrition Service, Bari–Metropolitan Area, Piazza Chiurlia 21, 70122 Bari, Italy; ezio.daniello@asl.bari.it (E.D.); matilde.gramegna@asl.bari.it (M.G.); alessandra.nencha@asl.bari.it (A.N.); 7 Prevention Department, Food Hygiene and Nutrition Service, Bari–North Area, Via De Chirico 23, 70056 Molfetta, Italy; antonio.caputo@asl.bari.it (A.C.); marta.giovine@asl.bari.it (M.G.); caterina.spinelli@asl.bari.it (C.S.)

**Keywords:** edible ice, public health, PAEs, heavy metals, SPME-GC/MS, ICP/MS

## Abstract

Edible ice is often produced by special machines that can represent a source of significant chemical and microbiological contamination. In this work, the presence of phthalic acid esters (phthalates, PAEs) and heavy metals in ice cubes distributed by 77 vending machines installed in two different zones in southern Italy and fed by water from the public water supply was investigated. Solid-phase microextraction coupled to gas chromatography−mass spectrometry (SPME-GC/MS) was used to evaluate contamination with four PAEs, which were selected because they are commonly used in the production of food-contact plastics, while inductively coupled plasma mass spectrometry (ICP/MS) was used to quantify the heavy metals. It was found that ice samples, especially those from one of the two considered zones (zone 2), exceeded the dibutyl phthalate (DBP) threshold limit value; some ice cubes from the other zone (zone 1) instead showed levels of both lead (Pb) and nickel (Ni) up to one order of magnitude higher than those observed in samples collected in zone 2 and higher than the maximum permitted values (European Directive n. 2184/2020). Since the water source connected to the ice vending machines was found to be free from significant levels of all considered target compounds and metals, the high levels of DBP, Ni, and Pb in ice cubes could be attributed to the components and/or to the state of repair of the ice vending machines themselves.

## 1. Introduction

Ice is commonly used as an ingredient or cooling agent in food and beverages, as well as for the preservation of food matrices, especially fish products. Edible ice is a valuable resource, especially during the summer months, being used in large quantities for a variety of purposes, including the preparation of cold drinks and the maintenance of the cold chain, which is essential to prevent the spoilage of perishable foods. However, data from the literature have demonstrated that ice can become contaminated with a range of both microbial organisms and chemical substances [1,2]. 

### 1.1. Phthalates (PAE) in Edible Ice from Vending Machines

Phthalic acid esters, known as phthalates (PAEs), are additives widely used by the polymer industry to make plastics flexible and soft at room temperature. 

Due to their accessibility, preservative properties, and low cost, plastic materials (e.g., polyethylene, PE; polyethylene terephthalate, PET; polypropylene, PP; etc.), are now widely used by the food and drinking-water industries [3,4,5]. 

They degrade under certain environmental and handling conditions and, therefore, can contaminate food or water through the migration of structural chemicals [6,7,8]. Studies on the integrity of plastics have demonstrated the migration of PAEs from PE at high density (HDPE) and from PET into food matrices and drinking water in contact with them. It has also been observed that PAEs are released earlier from HDPE than from PET materials containing drinking water [9,10]. The migration of PAEs from polymers is a complex process that depends on many factors such as their initial concentration in the plastic, the temperature, the nature of the food/water, and the contact time.

For humans, ingestion of contaminated food or water represents a major route of exposure to PAEs. Chronic exposure to PAEs can compromise neurological development [11], increase the risk of cardiovascular diseases [12] and cause renal, hepatic, and reproductive dysfunctions [13,14,15]. A relationship has also been theorized between asthma and other allergic disease conditions and exposure to phthalates in the diet [16].

Regulation (EU) No. 10/2011 provides guidelines for risk assessment with regard to the migration of substances from plastic to food [17], while Regulation (EU) No. 1907/2006 [18] regulates good manufacturing practices, allowing the release of PAEs at levels up to 10 mg of substance per 1 dm^2^ of material surface [17]. 

This work aims at the quantification in edible ice of four PAEs, namely, dimethyl phthalate (DMP), diethyl phthalate (DEP), dipropyl phthalate (DPP), and dibutyl phthalate (DBP), which were selected because they are commonly used as additives in the production of food-contact polymers and tend to migrate from plastics to food/water, having high polarity and low molecular weight [19]. 

DBP has been listed by the European Commission on endocrine disruption and all current regulations as a priority substance [18], and its use is prohibited in cosmetics and personal-care products [20,21] even though (FCM No. 157) it is one of the five PAEs allowed in plastic materials in contact with food [22]. The migration limit of DBP into food is ≤0.3 mg/kg [15], while the limit for total PAEs is set at ≤60 mg/kg of food product (10 mg/dm^2^ of material surface) [17].

### 1.2. Heavy Metals

Iron (Fe), manganese (Mn), copper (Cu), cadmium (Cd), mercury (Hg), nickel (Ni), and other elements with density greater than 4.5 g/cm^3^ are heavy metals and may be present in water intended for human consumption, where they pose a threat to human health due to their toxicity. Arsenic (As) is a nonmetal, but its toxicity and some of its properties are like those of heavy metals, so it is also classified as a heavy metal [23]. They can bioaccumulate in the human body (e.g., in lipids and the gastrointestinal system), causing cancer and other chronic and sub-chronic effects [24,25,26]. 

Natural processes and anthropogenic activities (e.g., industry) represent the main sources of environmental pollution by heavy metals [27]. Poorly treated industrial, domestic, and agricultural wastewater can be additional sources of heavy-metal pollution [28,29]. In addition, Pb and Hg can also reach the atmosphere due to traffic and industrial activities and enter water systems because of dry and wet deposition [30]. 

Over the last years, heavy-metal pollution in drinking water and its associated effects have been the objects of growing interest [31,32]. Regulatory agencies, such as the World Health Organization (WHO) and the U.S. Environmental Protection Agency (US EPA), as well as the European Union (EU) Directive on the quality of water for human consumption, have set maximum limits on the amounts of heavy metals allowed in drinking water to minimize the risks to human health due to the daily intake of drinking water contaminated by them [33]. Additionally, numerous technologies and protocols have been developed to help remove metals from water [34] but, despite significant advances, research is still needed to ensure the safety of drinking water. To date, in fact, no technique for the complete removal of all heavy metals from drinking water has been documented.

To the best of our knowledge, the chemical contamination of edible ice produced by vending machines has not been investigated yet, leaving unanswered questions on the potential risks to humans related to the presence of target chemicals and/or their increased concentration with respect to the recommended values. 

The aim of this study was to evaluate contamination with PAEs and heavy metals in ice cubes produced by vending machines and intended for consumption.

## 2. Materials and Methods

In this study, the contamination with PAEs (DBP, DEP, DMP, and DPP) and 18 heavy metals (Be, Al, Ti, V, Cr, Mn, Fe, Co, Ni, Cu, Zn, As, Se, Cd, Sn, Sb, Tl, and Pb) in edible ice was evaluated. 

The ice samples were produced by vending machines distributed in two different zones (zone 1; zone 2) located in the south of Italy and supplied with water from the public water supply. 

The quantification of PAEs was carried out by solid-phase microextraction coupled to gas chromatography−mass spectrometry (SPME/GC-MS) [35,36], while the heavy-metal contamination was investigated by inductively coupled plasma mass spectrometry (ICP/MS) [37].

The target chemicals to investigate were chosen due to their reported toxicological relevance, their potential presence in the drinking water [32,38,39,40,41,42], and, in the case of PAEs, their presence in many plastic components of the considered dispensers (e.g., tank, pipes, fittings, etc.).

### 2.1. Ice and Water Samples

From April to September 2023, in the Apulia region (Italy), samples of edible ice were collected from the storage compartments of 77 ice vending machines that were connected to the public water supply and operative in public and shared catering establishments, which were randomly selected among those that usually benefit from a greater influx of customers during the summer season. 

For each ice-making machine, 1 L of the filling water and 1 kg of ice cubes were sampled, placed in glass containers and glass bottles, respectively, and transported to the Laboratory of Hygiene of Environmental and Food–University of Bari (Italy) in a special isothermal refrigerator controlled at 4 °C. In particular, 38 ice samples and 38 water samples were taken from ice vending machines located in the province of Barletta-Andria-Trani (BT, zone 1), and 39 ice samples and 39 water samples were taken from Bari (BA, zone 2). Sampling was performed by the Environmental Health Officers of the Local Health Authorities.

### 2.2. Determination of PAEs Contamination in Ice Samples

#### 2.2.1. Chemicals

DBP, DEP, DMP, and DPP standards (purity > 99%) were purchased from Sigma Aldrich (St. Louis, Missouri, USA). Stock solutions (1 mg/mL) were prepared in sterile filtered ultrapure water (SFUW, Sigma Aldrich, St. Louis, Missouri, USA) with 20% (*w*/*v*) NaCl (Sigma Aldrich) and stored in glass vials at 8 °C. Working solutions, which were obtained daily by diluting stock solutions with SFUW, were stored at 8 °C until use [35,43].

#### 2.2.2. SPME-GC/MS Experimental Conditions

Each ice sample was melted at room temperature. Then, 1.5 mL of water was transferred into a 1.7 mL vial and NaCl (20% *w*/*v*) was added. Next, the solution was subjected to SPME-GC/MS analysis [35,43]. A polydimethylsiloxane/divinylbenzene fiber (PDMS/DVB, d_f_ 65 µm, Sigma-Aldrich, St. Louis, MO, USA) was employed for the direct immersion (DI)-SPME procedure. PAEs extraction was conducted under constant stirring for 20 min at 40 °C. To avoid a possible “memory effect”, after the desorption step and before the subsequent extraction, the fiber was kept at 200 °C for 30 min in the GC injector. The GC/MS system was a Finnigan TRACE GC ultra gas chromatograph (Thermo Fisher Scientific, Waltham, MA, USA) equipped with a split/splitless injector and interfaced to an ion trap MS (Finnigan Polaris Q, Thermo Fisher Scientific, Waltham, MA, USA). The capillary column was a Sigma Aldrich SPB-5 fused silica (30 m, 0.25 µm i.d., 0.25 µm film thickness) with helium (purity > 99.999%, Rivoira, Bari, Italy) as the carrier gas (flow rate 1 mL/min). The temperature of the transfer line was 220 °C, while the injector (splitless mode for 2 min) was kept at 270 °C [35]. The oven temperature program was as follows: 50 °C; ramp 1:10 °C/min from 50 °C to 260 °C; hold at 260 °C for 3 min. The mass spectrometer was operated in the electron impact positive ion mode (EI+), with the ion-source temperature set at 250 °C. The electron energy was 70 eV, and the filament current was 150 µA. The total ion current (TIC, *m*/*z* range 40–300) acquisition mode was used. Analytes were detected using extracted ion chromatograms (XICs) obtained in TIC mode [35]. To remove carry-over, fiber was always subjected to a second thermal desorption after each chromatographic run. 

During all experimental procedures, the use of plastic objects of any type (tips, containers, etc.) was always avoided. 

To validate the described method, calibration curves were obtained by analyzing PAE working solutions in the concentration range 0.1–100 ng/mL. The limits of detection (LOD) and limits of quantification (LOQ) were determined by LOD ≅ (3·sda)/b and LOQ ≅ (10·sda)/b, where sda is the standard deviation of the y-intercept and b is the slope of the regression line. The within-day (n = 3) and between-days (n = 3 over 5 days) percentage relative standard deviations (RSD %) were calculated at three levels (0.5, 2.5, and 5 µg/mL) by analyzing solutions prepared daily from the same working solutions, which were stored at 8 °C [35]. 

Each measurement was repeated in triplicate. 

### 2.3. Metals in Ice Samples

#### 2.3.1. Chemicals and Standard Solution

A multielement certified standard mix in 2% nitric acid (concentration 20 mg/L) was purchased from Chemical Research 2000 s.r.l. (Roma, Italy). Six multielement standard solutions (concentration level in the range 0.02–300 ppb) were prepared by dilution of the primary standard with 0.6% nitric acid solution. The internal standard (CPA Chem, Bogomilovo, Albany, NY, USA) contained 8 elements: scandium (Sc), germanium (Ge), rhodium (Rh), indium (In), terbium (Tb), holmium (Ho), lithium (Li), and bismuth (Bi) at 10 mg/L. It was used at 10 ppb in nitric acid at 0.6% *v*/*v*.

#### 2.3.2. Analysis in ICP-MS

Water solutions from ice samples were analyzed in an ICP-MS ICAP Q System (Thermo Fisher Scientific, Waltham, MA, USA). Each solution was diluted before injection at 1:30 with ultrapure water (Sigma Aldrich). The analysis sequences were performed in KED mode with argon nebulizer flow and plasma gas flow at 1 L/min and 18 L/min, respectively. Eighteen elements (Be, Al, Ti, V, Cr, Mn, Fe, Co, Ni, Cu, Zn, As, Se, Cd, Sn, Sb, Tl, and Pb) were quantified with six-point calibration curves using the multielement standard solutions. The obtained correlation coefficients (R^2^) were always greater than 0.999. LOD and LOQ values were in the range 0.06–2.03 and 0.20–6.70 ng/mL, respectively. The extraction recoveries varied from 88% to 110% for all considered metals.

## 3. Results and Discussion

This investigation is characterized by an element of novelty with respect to the existing scientific literature and aims to fill the knowledge gaps on the potential chemical contamination of edible ice produced by vending machines and to hypothesize on the possible contamination sources. 

**Figure 1:**
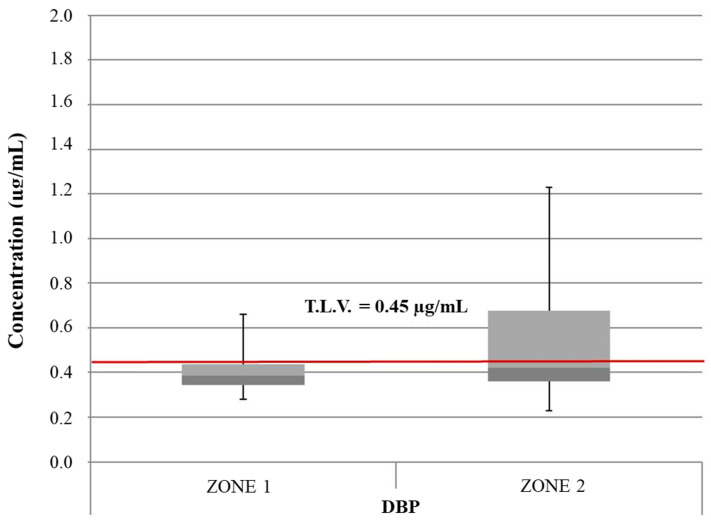
Box plot showing the concentrations of DBP in samples of edible ices from zone 1 and zone 2. Red line: threshold limit value (T.L.V.); dark gray: Q1; light gray: Q3.

From the complete analysis of all considered ice samples, 36 samples from zone 1 and all samples from zone 2 were contaminated with DEP at a concentration level of 0.08 ± 0.03 µg/mL. DMP was found in three samples from zone 1 and three samples from zone 2 (0.10 ± 0.03 µg/mL). No samples contained detectable amounts of DPP, while DBP was detected in all 77 considered ice samples at a broad range of concentrations, as shown in the box plot in Figure 1. 

For both sample populations (zone 1 and zone 2), outliers related to the exceedance of the T.L.V., which is equal to 0.45 μg/mL and was established by European Directive n. 2184/2020 [33], are shown. The variability in terms of DBP concentration is pronounced for ice samples collected in zone 2. Moreover, mean and maximum values for DBP in zone 2 ice samples (0.55 μg/mL and 1.27 μg/mL, respectively) are greater than those observed for ice samples collected in zone 1 (0.44 μg/mL and 0.70 μg/mL, respectively). DEP and DMP concentration levels were always within the limits permitted by current laws [44].

Considering that the analysis of the feed water of all ice dispensers revealed no appreciable contamination with the four PAEs of interest (concentration levels always lower than their respective LOQs) and taking into account that the water used for ice production is drawn directly by the distribution system and, therefore, from the plumbing system of the buildings where the ice machines are placed, the possibility that the contamination in terms of PAEs observed could be due to migration from the pipes of the water-distribution system could be excluded [45,46]. 

Possible sources of contamination include the plastic components of the ice vending machines themselves (tank, pipes, fittings, etc.), in which PAEs could be present as plasticizers added during their industrial manufacturing. Therefore, it was more likely that PAE migration into ice samples was related to the contact of the water with the polymeric container placed inside the ice machine for water storage. It was plausible, moreover, that the enrichment of PAEs in water and particularly the higher DBP concentrations observed in selected samples could be related to the permanence time of the water inside the polymeric container (e.g., stagnation), but no information regarding the frequency of water replacement inside each ice machine or the chemical composition of the plastic components of the machines themselves was available. Generally, long periods of stagnation of water inside the containers should be avoided. This finding is particularly alarming considering that the European Commission on endocrine disruption and all current regulations have listed DBP as a priority substance [18] and that its use is forbidden in cosmetics and personal-care products [20,21], even if (FCM No 157) it is one of the five PAEs allowed in plastic food-contact materials [22].

**Figure 2:**
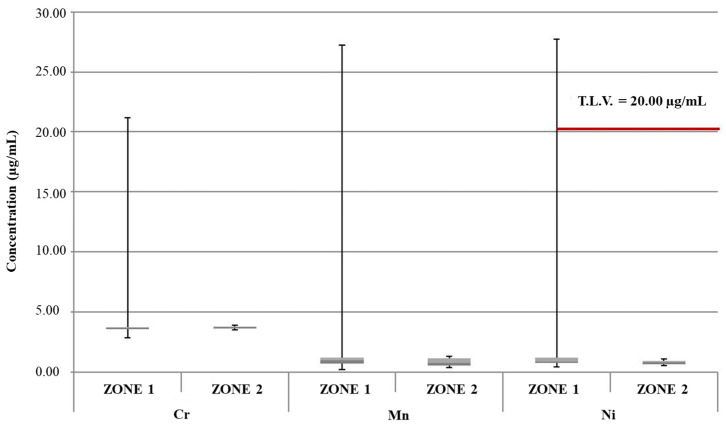
Box plot of concentrations of Cr, Mn, and Fe in groups of samples of samples of edible ice from zone 1 and zone 2. Red line: T.L.V.; dark gray: Q1; light gray: Q3.

Figure 2 shows a box plot representing concentrations of key heavy metals in samples of samples of edible ice collected from vending machines located in the two geographical zones under investigation in the present study (zone 1 and zone 2). The discussion herein focused on heavy metals subject to Italian regulations for drinkable water according to the Italian Legislative Decree n.18/2023 in the transposition of European Directive n. 2184/2020 [33]. Some of these heavy metals showed concentrations above their LOQs in the investigated samples. More specifically, experimental data were related to As, Cd, Pb, Cu, Ni, Se, Cr, Fe, Mn, V, and Al. In the graphs, moreover, the T.L.V. specific to each element is reported when that value was exceeded.

**Figure 3:**
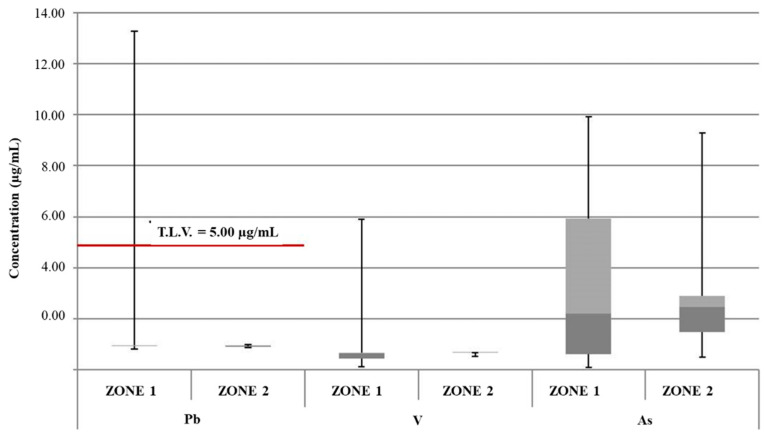
Box plot of concentrations of Pb, V, and As in groups of samples of edible ice from zone 1 and zone 2. Red line: T.L.V.; dark gray: Q1; light gray: Q3.

According to the legislation in force at the national and European levels, the established T.L.V.s are 20 μg/L and 5 μg/L for Ni and Pb, respectively [33,47]. With specific regard to the zone 1 samples, the aforementioned limit value for Ni was exceeded in 4 of 38 ice samples, with concentrations ranging from 21.08 μg/L to 27.7 μg/L (Figure 2). Additionally, an exceedance of the T.L.V. for Pb (concentration equal to 13.26 μg/L) was found in one ice sample from the zone 1 sample group; in the same sample, the Ni limit value was exceeded (concentration equal to 23.61 μg/L), suggesting a common contamination source (Figure 3). 

**Figure 4:**
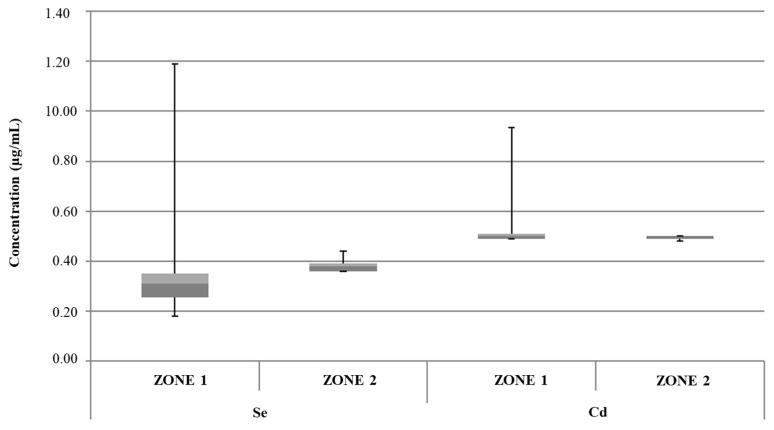
Box plot of concentrations of Se and Cd in samples of edible ice groups from zone 1 and zone 2. Dark gray: Q1; light gray: Q3.

**Figure 5:**
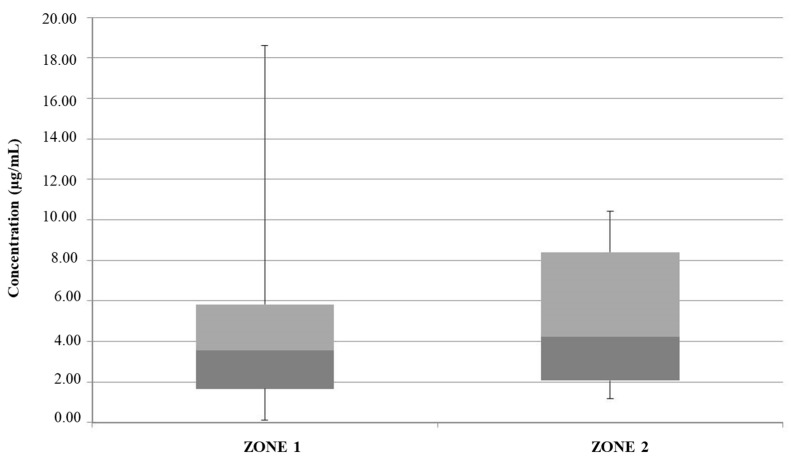
Box plot of concentrations of Al in samples of edible ice groups from zone 1 and zone 2. Dark gray: Q1; light gray: Q3.

**Figure 6:**
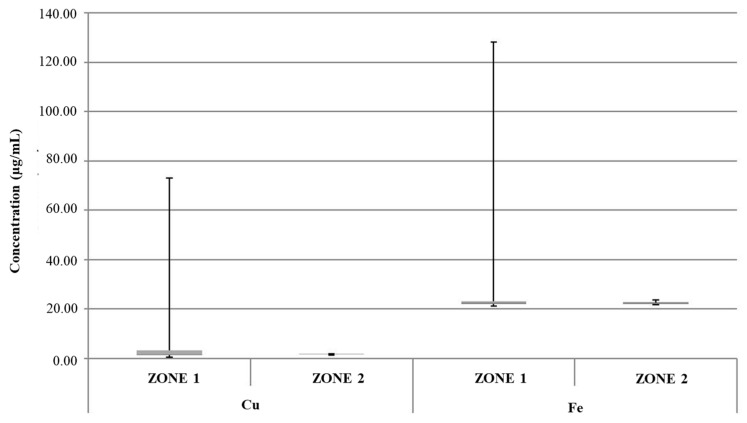
Box plot of concentrations of Cu and Fe in samples of edible ice groups from zone 1 and zone 2. Dark gray: Q1; light gray: Q3.

From the overall analysis of data reported in Figure 2, Figure 3, Figure 4, Figure 5 and Figure 6, it was possible to observe that the median value of both sample groups (zone 1 and zone 2) was, for all the investigated heavy metals (except Ni and Pb), up to two orders of magnitude below the specific T.L.V. Moreover, ice samples collected in zone 1 were characterized by variability in terms of concentration and peak values greater than those observed for samples from zone 2. 

Considering that the chemical composition in terms of regulated heavy metals upstream of the water-distribution system is subject to planned sampling and official controls by competent authorities and that the analysis of the feed water of all ice dispensers revealed no appreciable contamination with the considered heavy metals (concentration levels always lower than their respective LOQs), it is likely that the observed contamination in selected samples in terms of Ni and Pb concentrations higher than the limit values could be related to the presence of a specific source of contamination inside the ice vending machine itself.

Assuming that the observed contamination of ice samples with metals (e.g., reported cases of exceedances of EU limit values for Ni and Pb concentrations) could be related to the enrichment of these elements in water flowing along the pipelines inside the ice machines, a comparison with the concentration levels encountered in tap-water samples reported in selected studies in the literature was made (Table 1) [48,49,50,51,52].

Firstly, it must be highlighted that samples collected from zone 1 were characterized by mean values of Ni and Pb concentrations up to one order of magnitude higher than those observed in samples collected in zone 2. In addition, based on the comparison with the results of other studies, it appears clear that mean and maximum Ni concentrations (4.05 μg/L and 27.70 μg/L, respectively) in samples from 39 sampling points (ice machines) in zone 1 were higher than those reported in studies aimed at investigating metal contamination of tap water in Nigeria (maximum concentration equal to 0.46 μg/L) [51], in Iran (0.2 μg/L and 1.9 μg/L, respectively) [52], in India (1.07 μg/L and 2.73 μg/L, respectively) [50] and, most importantly, in a study aimed at determining the concentrations of key metals in water samples collected across the water-distribution system in Italy (1.02 μg/L and 2.44 μg/L, respectively) [49]. The only exception to underline, with respect to the overview herein reported (Table 1), is a study performed in Irbid governorate in Jordan [48], which shows mean and maximum Ni concentrations (16.8 μg/L and 517.3 μg/L, respectively) two orders of magnitude higher than those observed in zone 1 of the present study. With specific regard to Pb, the comparison revealed that mean and maximum concentrations found in the present study (1.26 μg/L and 13.26 μg/L, respectively–zone 1) were higher than those reported in Nigeria and Italy. Even if the several sampling points included in the study carried out in Italy do not cover the geographical area investigated in the present study, the findings confirmed that the heavy-metals contamination was probably linked to ice machines.

In the attempt to critically analyze the collected experimental data, it is relevant to mention that, throughout the literature, it is highlighted that differences in terms of contamination of drinking water with metals across various countries worldwide has to be considered to be the result of multiple factors, including the water source, the modality and typology of water sanitation and water purification, and the corrosion of materials used to cover the inner walls of water pipes or representing their main constituents. While the composition of the earth’s crust and dissolution of minerals into the water may have a direct effect on metal contamination, especially in terms of As, Mn and Se, pipes in the water-distribution system in urban settlements and/or building plumbing systems and/or ice-machine plumbing systems are responsible for the presence of specific metals such as Fe, Pb, Ni, Cu, and Zn and for the exceedance of their allowable concentrations in drinkable water as set by one or more regulatory limits [53]. Water contamination in terms of Fe, Pb, Ni, Cu, and Zn is mainly due to corrosion and may occur at several points along the plumbing system, including in metal and polyvinyl chloride pipes, pipe linings and coatings, solders, joints, and valves [53,54]. It has been recently documented that unplasticized polyvinyl chloride (PVC) pipes used in drinkable-water-distribution systems specifically leach Pb into the water [53,55,56]. Pb compounds are indeed used as stabilizers in the manufacturing of PVC pipes to enhance their strength and durability. Studies have also highlighted that Pb release from PVC pipes depends on physico-chemical properties of water such as pH and temperature and may be exacerbated in cases of water stagnation (e.g., time of exposure to the PVC material) [56]. 

In terms of public health, this investigation helps policy makers reflect on aspects of food safety that have potential repercussions for the general population and appreciate over time. Food-business operators must also provide their own contribution by adopting adequate practices to combat water stagnation in the system and promoting, at the same time, both proper sanitization and correct flushing; where possible, especially after long periods of inactivity of the machine, they should also check its state of repair.

Based on the above-reported observations and considering that no contamination with the investigated heavy metals was detected in the feed water for any of the ice dispensers, the high levels of key metals such as Ni and Pb found in the ice samples could be linked to their release from plastic tubes inside the ice vending machine. 

## 4. Conclusions

Ice vending machines could be a source of chemical and heavy-metal contamination in edible ice, as revealed by our investigation

SPME-GC/MS made it possible to highlight PAE contamination at levels that were in some cases higher than the T.L.V. fixed by the European Directive, while ICP/MS was useful in quantifying heavy metals; these were also sometimes present at levels higher than the maximum allowed levels, as in the case of Pb and Ni. 

The origin of the contamination was ascribed to the components of the machines themselves (e.g., tips, tubes, reservoirs, etc.), as the feed water always substantially free of the considered contaminants.

Therefore, this study raises the issue of the need to adopt proper mitigation strategies for the reduction of contamination in edible ice to prevent potential public-health consequences that can appreciate over time. These strategies should aim to promote, at the same time, both proper sanitization and correct flushing, especially after long periods of inactivity of the equipment, as well as frequent checks on its state of repair.

## Figures and Tables

**Table 1:** Concentrations of metals (mean value and range, µg/L) in tap-water samples (literature references) and in samples of edible ice investigated in this study. ND: not detected.

Mean Value Range (μg/L)	[49]	[47]	[50]	[48]	[46]	Study Zone 1	Study Zone 2
Al	/	/	/	44.99 (11.40–90.79)	/	4.31 (0.12–18.60)	5.13 (1.19–10.43)
V	/	/	/	/	/	0.92 (0.12–5.90)	0.66 (0.53–0.68)
Cr	(0.65–0.85)	0.34 (0.15–0.55)	25.0 (ND–78.0)	0.63 (0.06–2.45)	2.9 (0.1–19.8)	4.11 (2.86–21.15)	3.69 (3.50–3.87)
Mn	(0.51–0.80)	/	/	23.77 (0.79–217.46)	2.8 (ND–29.3)	2.90 (0.21–27.23)	0.86 (0.39–1.33)
Fe	(0.42–0.51)	/	28.0 (3.0–5.06)	58.74 (5.08–133.45)	156.2 (ND–4072)	25.67 (21.21–128.16)	22.47 (21.76–23.59)
Co	(0.34–0.44)	/	/	/	0.1 (ND–1.2)	0.48 (0.03–0.85)	0.53 (0.51–0.53)
Ni	(0.29–0.46)	1.02 (0.19–2.44)	0.2 (ND–1.9)	1.07 (0.11–2.73)	16.8 (0.2–517.3)	4.05 (0.45–27.70)	0.81 (0.52–1.11)
Cu	(0.23–0.26)	/	0.1 (ND–1.7)	5.48 (0.71–25.85)	43.5 (0.4–584)	8.49 (0.59–73.01)	1.75 (1.56–2.00)
Zn	(0.19–0.24)	/	13.0 (0.4–70.2)	241.22 (34.01–988.25)	244.8 (3.7–1849)	30.18 (2.44–291.89)	6.13 (5.25–7.55)
As	/	1.05 (0.54–1.58)	0.8 (0.4–1.1)	0.68 (ND–5.86)	0.5 (ND–3.3)	3.37 (0.11–9.93)	2.74 (0.49–9.28)
Se	/	/	/	/	/	0.35 (0.18–1.19)	0.38 (0.36–0.44)
Cd	/	0.92 (0.26–1.58)	1.0 (0.8–4.8)	/	0.1 (ND–0.7)	0.55 (0.49–0.94)	0.50 (0.48–0.50)
Pb	(0.31–0.39)	0.48 (0.26–0.58)	2.4 (0.9–9.4)	/	4.8 (ND–73.5)	1.26 (0.82–13.26)	0.93 (0.90–0.99)

## Data Availability

The original contributions presented in the study are included in the article and Supplementary Material, further inquiries can be directed to the corresponding authors.

## Supplementary Materials

The following are available online at https://www.mdpi.com/article/10.3390/foods13182910/s1, Table S1: Validation parameters of the analytical method for PAEs determination.
