# Supplementary material for: Analytical Investigation of Phthalates and Heavy Metals in Edible Ice from Vending Machines Connected to the Italian Water Supply
Source: Foods. 2024 Sep 13;13(18):2910. doi: 10.3390/foods13182910 (PMC11430881; doi:10.3390/foods13182910)
Supplement: Supplementary file 1 [file foods-13-02910-s001.zip › foods-3187697-supplementary.pdf]

Table S1. Validation parameters of the analytical method for PAEs determination

PAEs	slope	Correlation Coefficient	LOD (μg/mL)	LOQ (μg/mL)	Within-Day			Between-Day		
					Concentration levels (μg/mL)					
					**0.5**	**2.5**	**5.0**	**0.5**	**2.5**	**5.0**
DMP	$7x10^9$	0.9945	0.005	0.018	12.3%	12.6%	12.2%	26.8%	27.3%	26.9%
DEP	$7x10^9$	0.9903	0.006	0.024	14.8%	14.3%	14.6%	25.8%	25.1%	25.5%
DPP	$1x10^{10}$	0.9922	0.006	0.021	14.3%	14.9%	14.4%	21.9%	22.5%	22.0%
DBP	$6x10^7$	0.9915	0.058	0.200	25.5%	25.8%	24.9%	35.3%	35.7%	35.5%
